# Surface Magnetization Reversal of Wiegand Wire Measured by the Magneto-Optical Kerr Effect

**DOI:** 10.3390/ma14185417

**Published:** 2021-09-19

**Authors:** Tomoaki Nakamura, Hiroki Tanaka, Tomofumi Horiuchi, Tsutomu Yamada, Yasushi Takemura

**Affiliations:** Department of Electrical and Computer Engineering, Yokohama National University, Yokohama 240-8501, Japan; nakamura-tomoaki-vs@ynu.jp (T.N.); tanaka-hiroki-xs@ynu.jp (H.T.); mudapon@gmail.com (T.H.); yamada@ynu.ac.jp (T.Y.)

**Keywords:** Wiegand wire, Wiegand effect, magneto-optical Kerr effect (MOKE), magnetization reversal, large Barkhausen jump

## Abstract

The Wiegand wire is known to exhibit a unique feature of fast magnetization reversal in the magnetically soft region accompanied by a large Barkhausen jump. We clarified a significant difference between the magnetization reversals at the surface and at the entire cross section of a Wiegand wire. We conducted magnetization measurements based on the magneto-optical Kerr effect and applied conventional methods to determine the magnetization curves. The switching field of the magnetization reversal at the surface was greater than that at the initiation of a large Barkhausen jump. Our analysis suggests that the outer surface layer exhibits low coercivity.

## 1. Introduction

A large Barkhausen jump is a fast magnetization reversal typically observed in magnetic materials with bistable magnetization states [1,2,3,4,5]. Wiegand wires made of FeCoV alloy are optimum materials exhibiting this phenomenon [6,7,8]. A pulse voltage is induced in a pick-up coil wound around the wires during their fast magnetization reversal, which is known as the Wiegand effect [8,9]. The specific feature of the Wiegand wires is that the large Barkhausen jump is observed even for an applied magnetic field of slow changing rate. The amplitude and width of the induced pulse voltage do not depend on the frequency of the applied magnetic field. The pulse voltage of approximately 8 V can be obtained even for a slow movement of a permanent magnet [10], which is practically useful in sensor applications. Therefore, the Wiegand wires have been used in magnetic sensors, e.g., rotation and position sensors [10,11,12]. These sensors are operated without a battery supply, as they can self-generate a pulse voltage [13,14,15]. Additionally, they have recently attracted attention for applications in self-powered electronic modules [16,17,18].

A bistable magnetic structure is formed in FeCoV wires by twisting and annealing treatments. The magnetic properties of FeCoV wires have been reported in terms of annealing and torsion stress [19,20]. After the wires are twisted, their outer layers near the surface become magnetically soft with a low coercivity, whereas the center core remains magnetically hard with a high coercivity [20]. The coercive forces of the magnetically soft and hard regions are approximately 2 and 8 mT/*μ*_0_, respectively, although they depend on the preparation conditions and the length and diameter of the wires. Here, *μ*_0_ represents the permeability in vacuum. In fact, the coercive force in the wire is considered to gradually vary along the radial direction.

We prepared twisted FeCoV wires of various diameters by etching their outer surfaces and measured their magnetic properties. The surface layers exhibiting lower coercive force were removed by etching [21]. However, the radial distribution of the coercive force has not been clarified. Additionally, some papers referred to a magnetic soft core and a hard outer layer [22,23], although supporting data were not sufficiently presented in most cases.

The objective of this study was to evaluate the magnetization reversal at the surface and to elucidate the magnetic structure of twisted FeCoV wires. The reported magnetization curves of wires commonly measured using a vibrating sample magnetometer (VSM) and B-H tracer using a pick-up coil represent the magnetization process in the entire sample volume.

This paper describes the magnetization process at the surface of a Wiegand wire evaluated in terms of the magneto-optical Kerr effect (MOKE). We present clear evidence supporting the significant difference between the magnetization processes at the surface and on the entire cross-sectional area of the wire, which is the main contribution of this study.

## 2. Materials and Methods

**Figure 1:**
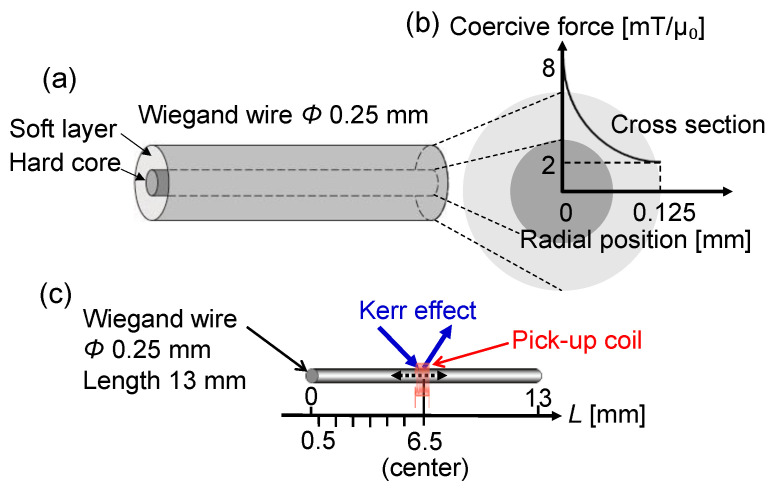
(**a**) Magnetic structure of a Wiegand wire and (**b**) distribution of the coercive force along the radial position of the wire. The outer layer and the inner core are assumed to have magnetically soft and hard properties, respectively; (**c**) setup of the magneto-optical Kerr effect (MOKE) and pick-up coil measurements for the Wiegand wire. The diameter and length of the wire are 0.25 mm and 13 mm, respectively.

A Wiegand wire with a length of 13 mm and a diameter of 0.25 mm was used in this study. The wire was made of Fe_0.4_Co_0.5_V_0.1_. The details of the wire have been previously reported [24,25]. Figure 1a,b show the magnetic structures of the wire used to interpret the measured magnetization properties of such wires [20,21]. In these figures, it is assumed that these structures have outer layers and the inner cores made of magnetically soft and hard materials, respectively. The magnetization curves of the wire were measured using a VSM. In this study, a uniform magnetic field along the length direction of the wire was applied for the VSM and MOKE measurements as well as for measuring the voltage induced in a pick-up coil.

**Figure 2:**
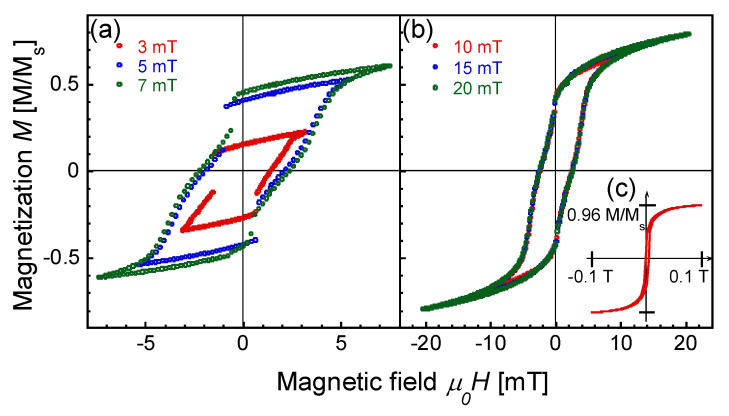
Magnetization curves of the Wiegand wire measured using a vibrating sample magnetometer. Minor loops traced under applied magnetic fields of (**a**) |*H*_ap_| = 3, 5, and 7 mT/*μ*_0_; (**b**) |*H*_ap_| = 10, 15, and 20 mT/*μ*_0_; and (**c**) a part of the full loop extracted in the field range of ±0.1 T/*μ*_0_ traced under an applied magnetic field of |*H*_ap_| = 0.3 T/*μ*_0_.

The minor loops, shown in Figure 2a,b, are the unique features of the Wiegand wire. When an alternating magnetic field in the range of |*H*_ap_| = 3 to 7 mT/*μ*_0_ is applied, the wire exhibits a fast magnetization reversal accompanied by a large Barkhausen jump. This switching field is constant at approximately ±1 mT/*μ*_0_ and independent of the applied field intensity *H*_ap_. When the intensity of the applied field is >7 mT/*μ*_0_, a fast magnetization reversal is not clearly observed, as shown in Figure 2b. In the applied field range of |*H*_ap_| = 10 to 20 mT/*μ*_0_ and higher, the magnetization curves are superimposed with a constant coercive force of 2.5 mT/*μ*_0_; the feature in this case is evidently different from that in the applied field range of |*H*_ap_| = 3 to 7 mT/*μ*_0_, where a large Barkhausen jump was induced. These fundamental properties of the magnetization process of the wire are in agreement with our previous measurement results [21,24].

Figure 2c, the insertion in the figure, shows a part of the major loop (from −0.1 to 0.1 T/*μ*_0_) traced under the maximum intensity of the applied magnetic field of *H*_ap_ = 0.3 T/*μ*_0_. The magnetization of the wire is 0.96 of *M*_s_ and almost saturates at 0.1 T/*μ*_0_, as shown in the figure, where *M*_s_ is the saturation magnetization.

To further analyze the magnetization process of the wire, the magnetization reversal at the surface was evaluated [26]. The MOKE of the wire was measured using commercial equipment (BH-753, NEOARK CORPORATION, Tokyo, Japan). A longitudinal MOKE setup was used. The magnetization along the wire, parallel to the direction of the applied magnetic field, was quantified by the change in the intensity of the polarized light coming from a white-colored light-emitting diode reflected at a surface area of 70 × 70 µm^2^. The magnetic domain was not clearly observed in this measurement, presumably because of the effects of the curvature and roughness of the wire surface. We avoided polishing or etching the wire surface not to modify the surface magnetization. Hence, the magnetic domain structure was not evaluated in this study. As shown in Figure 1c, the measurement area was positioned at *L* = 0.5–6.5 mm from the left end to the center of the wire.

## 3. Results and Discussion

**Figure 3:**
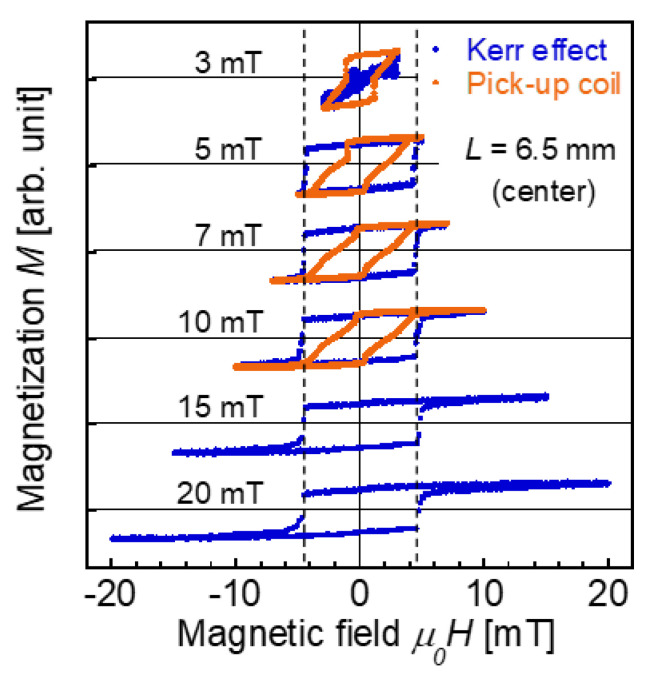
Magnetization curves of the Wiegand wire obtained from the MOKE and voltage induced in the pick-up coil. The measurement position is *L* = 6.5 mm, which is the center of the wire. An alternating magnetic field of |*H*_ap_| = 3 to 20 mT/*μ*_0_ is applied.

Figure 3 shows the magnetization curves obtained using the MOKE. The measurement position was *L* = 6.5 mm, which is the center of the wire. An alternating magnetic field of |*H*_ap_| = 3 to 20 mT/*μ*_0_ at 0.1 Hz was applied. The surface magnetization was reversed relatively steeply at approximately ±4.5 mT/*μ*_0_, independent of the applied field range of |*H*_ap_| = 5 to 20 mT/*μ*_0_.

The magnetization curve obtained from the voltage induced in the pick-up wound around the wire was also evaluated. As shown in Figure 1c, the position of the pick-up coil with 100 turns was varied, *L* = 0.5–6.5 mm, similar to the measurement area of the MOKE measurement. As the width (length along the wire direction) of the pick-up coil is 0.6 mm, the magnetization reversal at the position of the coil, not the entire length of the wire, contributes to the magnetization curves determined by this method [27]. An alternating magnetic field |*H*_ap_| = 3 to 10 mT/*μ*_0_ at 20 Hz was applied. The magnetization curves measured under this field frequency can be considered to represent a static magnetization process, which is equivalent to the magnetization process measured using the VSM and MOKE. The maximum intensity of *H*_ap_ was 10 mT/*μ*_0_ in this measurement, which was limited by the alternating current power supply used in the present study. The voltage induced in the pick-up coil was measured, which was proportional to $-\frac{d\Phi }{dt}$, which is the time differential of the magnetic flux *Φ* penetrating the cross section of the wire at the pick-up coil position [28,29].

Figure 3 shows the magnetization curves of the Wiegand wire obtained from the voltage induced in the pick-up coil. A large Barkhausen jump is clearly observed when *H*_ap_ is ±3 mT/*μ*_0_. This curve is quite similar to the minor loop of |*H*_ap_| = 3 mT/*μ*_0_ measured using the VSM, as shown in Figure 1b. The magnetization curves measured under |*H*_ap_| = 5, 7, and 10 mT/*μ*_0_, shown in Figure 3, are also similar to the minor loops measured by the VSM under the corresponding *H*_ap_ shown in Figure 2a,b. The difference between the magnetization curves measured by the pick-up coil and VSM is attributed to the difference in their measurement areas: locally at the coil position and along the entire length of the wire, respectively. The position dependence of the magnetization process is discussed later in detail.

Figure 3 shows the significant difference between the magnetization reversal at the surface and at the entire cross section of the wire. The surface magnetization is remarkably different from the magnetization measured by the VSM and pick-up coil. Here, we found that the switching field of 4.5 mT/*μ*_0_ at the surface does not match the switching field of 1 mT/*μ*_0_, initiating a large Barkhausen jump or magnetization reversal of the magnetically hard region of the wire. No increase in the magnetization was observed in the MOKE measurement after switching field of 4.5 mT/*μ*_0_ at the surface. As shown in Figure 1a,b, the magnetization at the applied field of 5 mT/*μ*_0_ was approximately 0.5 *M*/*M*_s_. The switching field for the half part of the wire that exhibits magnetically hard behavior is greater than 5 mT/*μ*_0_. Therefore, the magnetization reversal at the surface is not attributed to the magnetically hard region.

**Figure 4:**
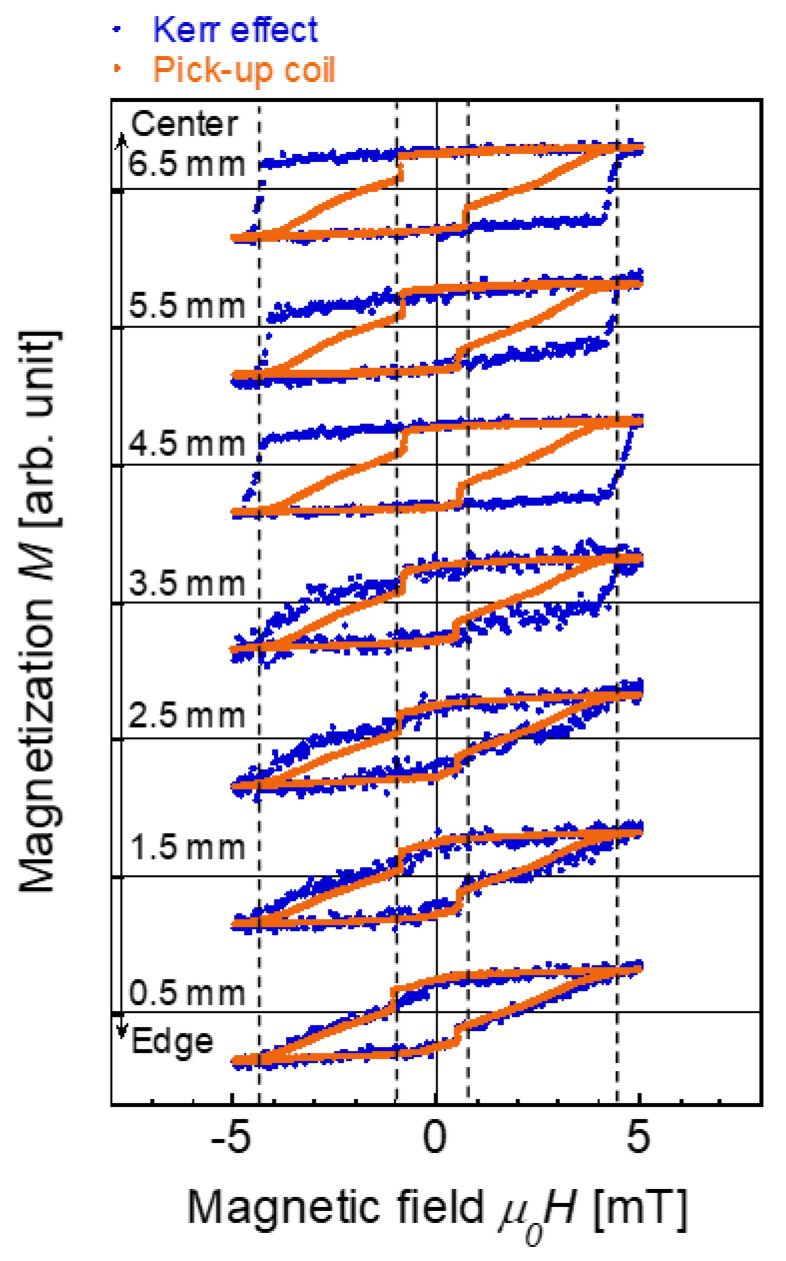
Magnetization curves of the Wiegand wire obtained from the MOKE and the voltage induced in the pick-up coil. The measurement position is varied at *L* = 0.5–6.5 mm of the wire. The alternating magnetic field of |*H*_ap_| = 5 mT/*μ*_0_ is applied.

To further investigate the surface magnetization, the magnetization process was evaluated depending on the position of the wire. Figure 4 shows the magnetization curves measured by the MOKE and pick-up coil. The measurement position was varied in the range of *L* = 0.5–6.5 mm. An alternating magnetic field of |*H*_ap_| = 5 mT/*μ*_0_ was applied. The magnetization curves measured at *L* = 3.5–6.5 mm by the MOKE were similar to each other with an equivalent coercive force of 4.5 mT/*μ*_0_. In this position range, a fast magnetization reversal was observed in the pick-up coil measurement and the magnetization curves obtained by the MOKE and pick-up coil were different from each other as previously discussed. When *L* = 0.5–2.5 mm, i.e., close to the edge of the wire, the coercive force measured by the MOKE was reduced to 1 mT/*μ*_0_. This magnetization reversal is considered to be a magnetically soft region at the surface. The magnetization curves obtained by the MOKE and pick-up coil showed a notable agreement. This match was only observed in the magnetization curves close to the wire edge, suggesting that the surface magnetization process dominates or is equivalent to the magnetization curve obtained by the pick-up coil affecting the magnetization of the entire cross section of the wire. A fast magnetization reversal was not clearly observed at *L* = 0.5 mm even in the pick-up coil measurement. This is attributed to the effect of the wire edge, mainly a strong demagnetizing field induced in the wire with a high degree of shape anisotropy [30].

An experiment to measure wires that are gradually etched from their surfaces to analyze the magnetization process depending on the radial position of the wire has been reported previously [21]. However, this method could not clarify the magnetic structure of the wires. This is because the magnetic interaction between the regions with different magnetization properties determines the magnetization process, particularly the large Barkhausen jump [31].

**Figure 5:**
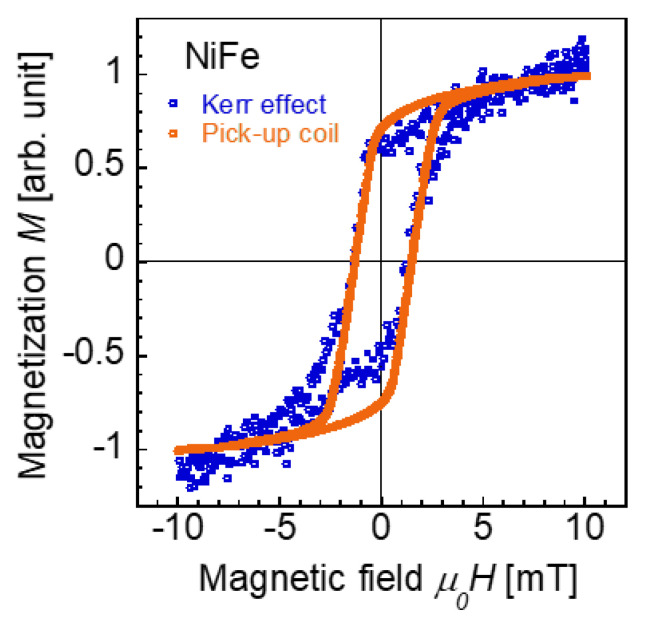
Magnetization curves of the NiFe wire obtained by the MOKE and the voltage induced in the pick-up coil. The diameter and length of the wire are 0.25 mm and 20 mm, respectively. The measurement position is at the center of the wire. An alternating magnetic field of |*H*_ap_| = 10 mT/*μ*_0_ is applied.

We confirmed the validity of the comparison in our measurements of the magnetization curves obtained by MOKE and from the voltage induced in the pick-up coil. Figure 5 shows the magnetization curves of the Ni_0.9_Fe_0.1_ wire with a diameter of 0.25 mm and a length of 20 mm obtained by these measurement methods. The applied magnetic field intensity was |*H*_ap_| = 10 mT/*μ*_0_. We used the same pick-up coil, excitation coils, equipment, and method throughout the measurements of the Wiegand and NiFe wires. The measurement area for both the MOKE and pick-up coil measurements was positioned at the center of the NiFe wire. As shown in the figure, the MOKE and pick-up coil measurements yield similar magnetization curves for the NiFe wire with a coercive force of *H*_c_ = 1.8 mT/*μ*_0_. The magnetization curves do not seem to be saturated, particularly that obtained by MOKE. This is presumably due to the rough surface of the NiFe wire, which diffuses the polarization of the reflected light at the surface.

## 4. Conclusions

The magnetization at the surface of a Wiegand wire was clarified by measurements using MOKE. We found that the surface magnetization exhibited a relatively steep magnetization reversal and that its switching magnetic field is evidently greater than that in the case of a magnetization reversal accompanied by the large Barkhausen jump. We clarified a significant difference between the magnetization curves obtained by the MOKE and the conventional VSM or pick-up coil measurement. The magnetization processes at the surface and at the entire cross section coincided with each other at the wire-length position close to the end of the wire. Considering that the magnetic structure of etched wires is difficult to analyze, the detailed measurements and analysis conducted in this study provide a new basis for understanding the magnetization process of Wiegand wires.
